# The active spread of adaptive variation for reef resilience

**DOI:** 10.1002/ece3.5616

**Published:** 2019-09-02

**Authors:** Kate M. Quigley, Line K. Bay, Madeleine J. H. van Oppen

**Affiliations:** ^1^ Australian Institute of Marine Science Townsville Qld Australia; ^2^ School of BioSciences The University of Melbourne Parkville Vic. Australia

**Keywords:** adaptation, assisted gene flow, climate change, coral reefs, restoration, thermal tolerance

## Abstract

The speed at which species adapt depends partly on the rates of beneficial adaptation generation and how quickly they spread within and among populations. Natural rates of adaptation of corals may not be able to keep pace with climate warming. Several interventions have been proposed to fast‐track thermal adaptation, including the intentional translocation of warm‐adapted adults or their offspring (assisted gene flow, AGF) and the ex situ crossing of warm‐adapted corals with conspecifics from cooler reefs (hybridization or selective breeding) and field deployment of those offspring. The introgression of temperature tolerance loci into the genomic background of cooler‐environment corals aims to facilitate adaptation to warming while maintaining fitness under local conditions. Here we use research on selective sweeps and connectivity to understand the spread of adaptive variants as it applies to AGF on the Great Barrier Reef (GBR), focusing on the genus *Acropora*. Using larval biophysical dispersal modeling, we estimate levels of natural connectivity in warm‐adapted northern corals. We then model the spread of adaptive variants from single and multiple reefs and assess if the natural and assisted spread of adaptive variants will occur fast enough to prepare receiving central and southern populations given current rates of warming. We also estimate fixation rates and spatial extent of fixation under multiple release scenarios to inform intervention design. Our results suggest that thermal tolerance is unlikely to spread beyond northern reefs to the central and southern GBR without intervention, and if it does, 30+ generations are needed for adaptive gene variants to reach fixation even under multiple release scenarios. We argue that if translocation, breeding, and reseeding risks are managed, AGF using multiple release reefs can be beneficial for the restoration of coral populations. These interventions should be considered in addition to conventional management and accompanied by strong mitigation of CO_2_ emissions.

## Graphical abstract

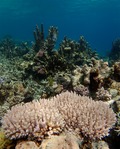

## 1 INTRODUCTION

The increasing pace and severity of environmental change has degraded ecosystems around the world and heightened the need for management interventions to support adaptation or restoration of species (Lindenmayer, Piggott, & Wintle, 2013; van Oppen, Oliver, Putnam, & Gates, 2015; Rinkevich, 2014). Coral reefs are sensitive to the effects of climate change and have declined globally, with even well‐managed reefs in the northern and central Great Barrier Reef (GBR) suffering unprecedented mortality due to heat stress in 2016 and 2017 (Hughes et al., 2018, 2017; Stuart‐Smith, Brown, Ceccarelli, & Edgar, 2018). On coral reefs, restoration efforts have so far primarily aimed to increase local abundance of individual species with stock from wild populations or nurseries but active interventions focussed around adaptation are increasingly being proposed (Anthony et al., 2017). These interventions have inherent risks and should therefore only be considered after due processes that incorporate a design tree framework for planning for success and failures (IUCN, 2013).

The genetics of source material is considered mostly in relation to the maintenance of genetic diversity (Baums, 2008) and has thus far rarely been explicitly addressed in the management of adaptive processes (Edwards, 2010). Genetic diversity underpins the potential scope for adaptation of natural populations, and its maintenance is a key objective for biodiversity management (Frankham et al., 2017). Genetic diversity describes the level of variation in genetic regions (polymorphic loci) and among individuals within populations. Beneficial adaptations are generated through mutation and recombination, while changes in their frequencies occur through natural selection and drift. Adaptive diversity is a subset of total genetic diversity and describes variants of genes that are directly associated with tolerance and survival (adaptive loci; Ahrens et al., 2018). Standing genetic variation is typically high in corals and has been demonstrated in resilient populations from highly variable environments or in survivors of bleaching (Palumbi, Barshis, Traylor‐Knowles, & Bay, 2014). While there are a number of candidate adaptive loci (Kenkel et al., 2014), and particularly those loci correlated with thermal tolerance (Dixon et al., 2015; Dziedzic, Elder, Tavalire, & Meyer, 2019; Jin et al., 2016), more work is needed to ground truth these putatively adaptive loci (PALs).

Ecological genomic and quantitative genetic theory has been proposed as a way to develop restoration methods that go beyond maintaining genetic diversity and aims to increase resilience to future environmental pressures (Baums, 2008; van Oppen et al., 2015). Such methods include selective breeding, conditioning (e.g., stress hardening via exposure to sublethal elevated temperatures), and microbial manipulations, and are collectively termed assisted evolution (van Oppen et al., 2015). Variation in thermal tolerance of scleractinian corals is underpinned by both acclimatory (i.e., phenotypic plasticity) and genetic mechanisms. Partial acclimation to heat stress has been shown to be possible within the life span of a coral colony and over relatively short periods of time (Bay & Palumbi, 2015), mediated by rapid changes in gene expression (Barshis et al., 2013; Kenkel & Matz, 2016) and microRNAs (Gajigan & Conaco, 2017). While this provides promise for conditioning as an intervention tool, the extent to which acclimation is heritable from one generation to the next remains to be shown. The potential for and rate at which corals can respond to ocean warming depend on the genetic architecture of thermal tolerance—a characteristic that appears to be variable among coral species. Genetic thermal tolerance can manifest as a heritable, polygenic trait involving over 100 loci of small effect sizes each (Bay & Palumbi, 2014; Dixon et al., 2015) or a smaller number of loci with larger effect sizes (Jin et al., 2016). Although the genetic architecture may be complex, efforts targeted at influencing coral host genetics provide avenues for assisted evolution (van Oppen et al., 2015).

Translocation describes the human‐mediated movement of individuals within established ranges or extant habitat, and in the more extreme case, movement beyond current ranges (assisted colonization/migration; Aitken & Whitlock, 2013; Kelly & Phillips, 2016; Seddon, 2010; Weeks et al., 2011). Assisted gene flow involves the intentional translocation of individuals with PALs into populations with an absence or low prevalence of these genetic variants to facilitate adaptation to new or anticipated local conditions (Aitken & Whitlock, 2013). As a variation on AGF sensu stricto, warm‐adapted corals harboring PALs may be selectively bred or crossed with cooler‐adapted conspecifics ex situ, then deployed at the cooler but warming locations. This intervention strategy may result in the introgression of heat tolerance alleles into the genomic background of the receiving population, thereby preparing those populations for further climate warming. Given that central corals transplanted southwards may bleach and potentially die when exposed to cooler winter temperatures in the south (Howells, Berkelmans, van Oppen, Willis, & Bay, 2013; van Oppen, Puill‐Stephan, Lundgren, De'ath, & Bay, 2014), selective breeding between northern corals with central and southern corals may alleviate this trade‐off.

Phenotypic variation in heat tolerance exists within and among populations and this is likely underpinned by genotypic variation, leading some individuals to survive a heat wave while others do not. Despite the comparatively high thermal tolerance of northern GBR corals (Dixon et al., 2015), the extreme heat waves in 2016 and 2017 caused extensive coral mortality (Hughes et al., 2018, 2017; Stuart‐Smith et al., 2018). However, surviving corals in northern populations could serve as a source of adaptive genetic variation for thermal tolerance and used in active interventions (Pardo‐Diaz et al., 2012; Tigano & Friesen, 2016). Indeed, AGF and the selection of thermally tolerant genetic stock were proposed for the management of corals reefs over ten years ago (Hoegh‐Guldberg et al., 2008). While it is likely that wild populations are already responding to the strong natural selection of bleaching events, AGF has the potential to accelerate the rate at which this occurs and therefore reduce the time needed for adaptive change. Given the rapid rate of coral loss on the Great Barrier Reef, feasibility assessments of proposed interventions are needed. The models presented here provide preliminary quantitative estimates as to the feasibility (scale and time frame) of applying AGF while also outlining model limitations, and thereby provide a clear path and scope for future research.

The effect of AGF and selective breeding may be persistent in longer living and annually reproducing species long after the initial deployment of stock. In this scenario, translocated individuals may continue to add PALs into receiving populations throughout their reproductive life to support natural reproductive and recovery processes even after initial deployment of coral stock. By producing coral larvae or juveniles with PALs that eventually reach a size/age of reproduction, these corals become part of the reef community and therefore the natural recovery process through continual supplementation of PALs to local populations (Vallee, 2004). Spatial scales for many existing restoration efforts are generally square kilometers, however, restoration of many species is needed on a much larger scale given the large extent of coral bleaching and mortality (Hughes et al., 2017). AGF differs from natural gene flow in that it is a directed pulse of individuals with PALs (single or multiple) and can thus encompass much larger distribution areas compared to natural gene flow (Aitken & Whitlock, 2013; Kelly & Phillips, 2016). The selective breeding of individuals affords the opportunity to increase the prevalence of PALs as well as increase the production scale of individuals with those adaptive variants. Hence, such restoration and adaptation efforts may scale‐up to spatial scales better able to influence regional reef scale degradation, although this may require several generations to achieve.

## 2 IS THE NATURAL RATE OF SOUTHWARD GENE FLOW OF PUTATIVE ADAPTIVE LOCI FROM THE NORTHERN GBR SUFFICIENT TO PREPARE COOLER REEFS FOR FURTHER WARMING?

Gene flow naturally spreads adaptive variants. Measuring levels of gene flow can inform the potential for PALs to penetrate throughout a species range and allows assessment of the efficacy of AGF and selective breeding to fast‐track adaptation. The success of the assisted spread of PALs beyond the primary deployment sites will also be influenced by the extent and direction of natural connectivity. Contrary to initial assumptions of complete connectivity in marine systems, corals vary in their dispersal ability and genetic connectivity (reviewed in Jones et al., 2009; van Oppen & Gates, 2006). Many coral species are highly differentiated with low levels of gene flow (Lukoschek, Cross, Torda, Zimmerman, & Willis, 2013; van Oppen, Bongaerts, Underwood, Peplow, & Cooper, 2011; Underwood, Smith, Oppen, & Gilmour, 2009; Warner, Oppen, & Willis, 2015), as initially described in early studies on predominantly brooding species on the GBR (Ayre & Hughes, 2004). Regional differentiation has also been detected in a number of species from the Caribbean, the Red Sea, and Western Australia (Baums, Johnson, Devlin‐Durante, & Miller, 2010; Devlin‐Durante & Baums, 2017; Drury, Manzello, & Lirman, 2017; Drury, Schopmeyer, et al., 2017; Foster et al., 2012; Howells, Abrego, Meyer, Kirk, & Burt, 2016; Rippe et al., 2017; Underwood, Richards, Berry, & Gilmour, 2017). Estimates of model‐based connectivity suggest some dispersal occurs between major regions on the GBR (Treml & Halpin, 2012) and has been supported by further genetic and modeling data showing some acroporid coral species exhibit low differentiation (Lukoschek, Riginos, & Oppen, 2016; Matz, Treml, Aglyamova, & Bay, 2018; van Oppen, Bongaerts, et al., 2011; Shinzato, Mungpakdee, Arakaki, & Satoh, 2015).

Together, this evidence supports that some level of gene flow occurs and thus that natural penetrance of PALs is possible. It is important to consider that these gene flow estimates encompass several generations or longer ecological time scales, which likely involve a stepwise process of spread over 1,000s of km. This contrasts with the relatively short time frame (30–80 years) to an estimated increase of 1–2°C above pre‐industrial temperatures (IPCC, 2007). By 2070, annual heat waves are projected to occur on >75% of reefs (van Hooidonk et al., 2016) even if dramatic warming globally is abated. Therefore, even if natural connectivity is extensive, AGF may be required to accelerate the spread of PALs. Given patterns in realized dispersal vary by species and potentially by region, research is needed to determine where donor colonies with PALs will need to be sourced from and where they will need to be placed. Additionally, estimates of rates of fixation are also needed to assess the feasibility of AGF and selective breeding as tools for assisting PAL spread.

## 3 MATERIALS AND METHODS

Temperatures across the GBR follow a north‐south pattern, where waters are warmer in the north and cooler in the south. The northern GBR encompasses >5° of latitude and has experienced the least overall warming from 1985–2012 compared to other regions on the GBR (predominantly −0.2 to 0 decrease in °C/decade), whereas the smaller central (<4°) and southern regions (~4.5°) have increased by 0.8°C and inshore, southern areas have cooled overall (Heron, Maynard, van Hooidonk, & Eakin, 2016). Evidence of adaptation to local thermal regimes across this latitudinal gradient exists (Dixon et al., 2015; Jin et al., 2016; Jurriaans & Hoogenboom, 2019; Lundgren, Vera, Peplow, Manel, & Oppen, 2013; Woolsey, Keith, Byrne, Schmidt‐Roach, & Baird, 2015), and northern populations may therefore serve as a source of thermal tolerance PALs for reefs further south.

### 3.1 Biophysical models

**Figure 1:**
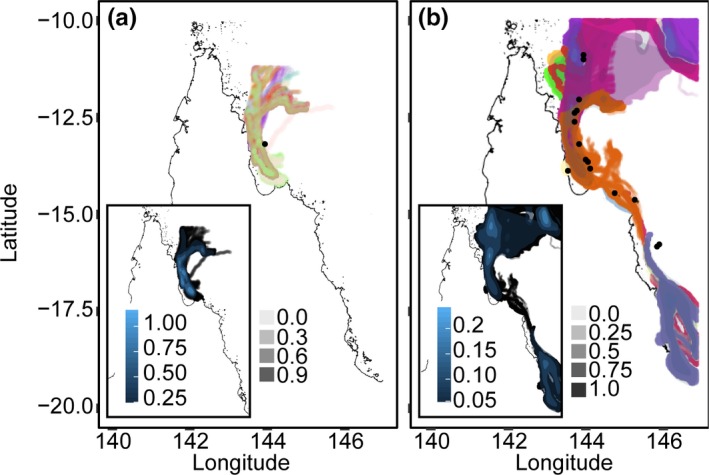
Connie II oceanographic larval dispersal modeling from single (a, inset, black dot corresponds to Tijou reef) and 15 larval release sites (b, inset, multiple black dots). Colors correspond to either replicate dispersal runs for the single reef (a) or for runs for different reefs (b, replicates not shown for clarity). Shading per color (as depicted by lighter to darker color shading) corresponds to calculated probabilities of larvae being in particular locations. Blue shading within insets corresponds to accumulated probabilities of larvae being in particular locations across different replicate runs and different reefs. Dark blue corresponds to locations with the highest accumulated probabilities of larvae (insets within a and b)

Larval connectivity models were used to investigate if barriers for larval dispersal exist between the warmer northern GBR and the cooler central and southern regions. These models were run using a single larval release site (using the warm northern Tijou Reef as an example, Figure 1a) or as multiple reefs (15 in total from the northern region) with similar biophysical parameters (Figure 1b), including three previously identified as key source reefs (Hock et al., 2017). Biophysical larval dispersal models were run in Connie2 using the Great Barrier Reef larval connectivity model at 1 m depth (version date range: 02/11/2008–30/03/2009). The Connie2 models use real ocean current data to model the movement of water between reefs at different depths. Larval connectivity was modeled during peak spawning times (both days and month), with simulated larvae released from November 15 19:00 to November 15 20:50, 2008 over 25 replicate runs originating from Tijou Reef (−13.177047, 143.949718). Larval dispersal time (i.e., pelagic larval duration‐PLD) was set at 96 days and 0 hr (Table S1). Dispersal potential was determined from larval duration and calculated as an average of the larval survival longevity of eight *Acropora* species (Graham, Baird, & Connolly, 2008).

To explore the geographic spread of PALs using dispersal parameters for multiple release sites, 10 additional reefs were selected that had similar biophysical characteristics using the eReefs Hydrodynamic models (for a total of 15 release sites). Specifically, reefs in the Cape York region in 2017 that had similar average annual temperature (average ~27°C) and ocean current speed profiles (~0.267 m/s), both calculated at a 1.5 m depth, were selected. Reefs were excluded if they fell below 29°C as a mean midsummer month SST (Matz et al., 2018). One additional site was selected (Wilkie Reef; −13.773711, 143.642835) due to the known presence of adaptive variants involved in thermal tolerance (Dixon et al., 2015). Finally, three other reefs that fit these criteria were selected because they were identified as the only robust sources of larvae in the Far North (Hock1: −11.018, 144.0131; Hock2: −11.1218, 144.0055; Hock3: −13.6756, 144.1688; Hock et al., 2017). Connie2 was re‐run with the same parameters as described for the single reef model.

### 3.2 Rate and extend of PAL fixation

To estimate how fast PAL fixation could spread using a linear stepping‐stone model (Slatkin, 1976), a rounded selection coefficient (*s*) of 0.05 was used (exact estimate calculated was 0.047), derived from the average values of 96 linear differential selection gradients reviewed in Morjan and Rieseberg (2004). All migrants were assumed to be homozygous, and fixation in these models was defined as the point at which a single allele became homozygous for all members in a population, for example, when the frequency of “AA” becomes fixed over “Aa” or “aa.” A second selection coefficient (0.1) was chosen as a larger value for selective advantage of mutant variants (*s*) given it was found as an average value in plants (0.11, Rieseberg & Burke, 2001) and through other methods using animal data (0.13, Morjan & Rieseberg, 2004). Both values are within ranges previously modeled by Slatkin (1976). Note that a selection coefficient of 0.05 may represent an under/overestimate given that these measurements do not include survival under thermal stress.

To estimate effective population size of migrants, *N*_m_ was estimated using genetic estimates from *Acropora tenuis* and *Pocillpora damicornis*. One to seven first‐generation migrants (FGMs) were estimated explicitly per site for *A. tenuis* (Lukoschek et al., 2016), but were calculated as the number of migrants per population (3.65 and 5.20) for two *P. damicornis* morphs (Torda, Lundgren, Willis, & Oppen, 2013). These values were then averaged to a value of ~4 migrants per generation. Specifically, these models assumed that these four migrants moved from one population to the next (unidirectionally).

To estimate average interpopulation reef distances, the distance between each reef and every other reef on the GBR was calculated, where each reef was given a reef centroid position. Average distances between reefs were then calculated per region (north, central, south). These distances were converted to rates using the Slatkin models. At a rate of 0.077 (north), 0.102 (central) and 0.067 (south) km/generation (*s* = 0.1), distances from Tijou reef (northern), Backnumbers reef (central), and Heron Island (south) as epicenters were calculated for the following timescales: 32, 50, 100, 500, and 1,000 years after PAL release. These timescales correspond to IPCC representative concentration pathways estimates, where 32 years from 2018 corresponds to the 2050 + 1°C Scenario, and ~82 years from present corresponds to the 2100 IPCC + 2°C Scenario (IPCC, 2007).

## 4 RESULTS

The spread of larvae was variable across the replicate release events from a single reef in the northern part of the dispersal envelopes (Figure 1a). The fan effect in the northern extent may be caused by variability in southeasterly trade winds or eddies and tidal jets within reefs generated by changes in flow along the shelf‐edge and the North Queensland and Gulf of Papua Currents. These winds and currents vary along multiple timescales (within days, seasonally, and interannually), and particularly within the 96 days of modeled larval dispersal (e.g., reversal and increase variability of tradewinds in December; Johnson et al., 2018; Ridgway, Benthuysen, & Steinberg, 2018). These factors are potentially coupled with the inherent variability associated with modeling the shallow, complex bathymetry of coral reef habitats (Bode, Bode, Choukroun, James, & Mason, 2018). The highest probability density of dispersed larvae was retained within ~1.25° north and south of the release reef (light blue peaks) in all replicate releases from a single reef. When a more realistic multiple larval release model was run, dispersal was predictably greater (Figure 1b), in which the extent of larval movement south depended on larval release reef location. Even when multiple release reefs were simulated, however, the vast majority of larvae were retained within the area between −10° and −11.25° north on the GBR (Figure 1b inset).

Dispersal modeling over four years of spawning events suggests that relatively few reefs contribute to the connectivity on the GBR across large spatial scales, with as little as 3% of reefs reaching ~47% of the GBR in a single reproductive event (Hock et al., 2017). Unbleached reefs predominantly located in the central and southern GBR can repopulate northern reefs against the prevailing current and ~80% of reefs that were classified as robust sources of larvae showed high local retention (Hock et al., 2017). Even highly dispersive species like *Acropora millepora* exhibited high localized retention after a four‐week simulation, with other species showing increased local retention compared to *A. millepora* (Thomas et al., 2014). Genetic connectivity models using larval dispersal kernels suggest that some mixing occurs across regions of the GBR over multigenerational timescales (Lukoschek et al., 2016), although this may be highly dependent on year (Torda et al., 2013). Our preliminary results confirm that local retention is high in the north. Although only a few larvae may be important for genetic connectivity, all of these results remain untested and may overestimate connectivity given the small number of larvae considered to constitute demographic “connectivity” (e.g., 1 larva/dispersal event).

Estimates of natural levels and directions of gene flow can inform the design of optimal deployment of PALs through AGF or deployment of offspring from crosses between northern and more southern corals (Harrisson et al., 2016; Pavlova et al., 2017). Estimates of gene flow will also help inform how fast PALs may spread naturally following intervention, thereby providing guidance for the number and location of reefs where migrants harboring PALs can be best deployed. The deployment of corals with PALs on well‐connected reefs facilitates a faster spread of PALs than if placed in isolated populations, as demonstrated by selective sweeps (Pialek & Barton, 1997). Understanding natural levels of population connectivity (realized dispersal) and source and sink dynamics is therefore key to the design and success of AGF and deployment of selectively bred coral stock. Results presented here from both single and multiple release scenarios demonstrate that there is value in the translocation of northern PALs into central and southern GBR reefs given the propensity for heat‐adapted larvae to naturally be retained within the northern GBR sector. Further modeling should assess where central and southern release sites should be placed to maximize PAL spread and fixation.

### 4.1 Limitations of biophysical models

The current resolution of biophysical models may not yet be fine enough to resolve individual reef scale differences and should therefore be taken with caution when formulating specific management decisions (Bode et al., 2018). A further limitation is that these models only consider one year of oceanographic data (2008), although there is evidence that subtidal flows (time scales longer than tidal fluctuations) are persistent and stable across longer time scales (e.g., >8 years) and depths (Ridgway et al., 2018). Additional modeling should simulate larval dispersal under variable, large‐scale oceanographic conditions.

We have made further simplifying assumptions in our models and acknowledge that key information has not yet been incorporated (e.g., temporal and spatial variability in larval competency). A daily percentage of larval mortality and competency was not incorporated, and we therefore assumed constant larval mortality and gain/loss of larval competence (Connolly & Baird, 2010; Treml & Halpin, 2012). This biophysical modeling therefore relies completely on PLD and assumes that dispersal is predominantly determined by biophysical, “mesoscale” processes like surface currents. Larval output as a function of adult fecundity and density at each site was also not parameterized as we were only interested in the presence/absence of larvae reaching different reefs and not the actual number reaching each reef, which was addressed in later models. Furthermore, these models are biased toward fast‐growing *Acropora* species and would need to be re‐run across a diversity of species with different life history characteristics to obtain more broad‐scale insights into dispersal potential on the GBR. Given these general caveats around biophysical models and specific parameterization, we interpret our results at the regional level, and combined, they suggest that the southern movement of potential PALs from the far northern GBR is naturally limited.

### 4.2 Using selective sweep theory to estimate the potential for assisted gene flow

Selective sweep theory can be used to quantify the rate of spread of gene variants and can thus be applied to predict the success of AGF, selective breeding, and guide optimal deployment designs. Selective sweeps occur naturally when advantageous alleles increase in frequency in a population over time. A hard selective sweep refers to complete fixation (i.e., complete dominance of a single gene variant), but sweeps can also be soft and result in the maintenance of alternative variants and hence greater diversity than hard sweeps. Sweeps occur relatively frequently in nature and tend to be common in species with large dispersal potential (Ralph & Coop, 2010). They can occur when a mobile population encounters new environments or alternatively, when the environment changes around populations of species with sedentary adults, such as corals. If selection on the beneficial variants is strong, those variants can spread rapidly and fix across metapopulations even under low connectivity (Slatkin, 1976; Tigano & Friesen, 2016). High levels of standing genetic variation will increase sweep success due to the increased chance of advantageous alleles to reach high frequencies (Aitken & Whitlock, 2013) by diminishing the impact of genetic drift (Hereford, 2009). Encouragingly, high levels of standing genetic variation exist in corals (Palumbi et al., 2014). Temperatures are already changing in ecologically meaningful ways, with bleaching occurring more frequently (Hughes et al., 2018), which suggests that selection pressure for heat tolerance should increase. Theory can therefore be used to provide potential conditions and designs under which successful selective sweeps can occur.

### 4.3 Spatial extent of penetrance of putative adaptive loci

Dispersal and migration are not equivalent to gene flow (Tigano & Friesen, 2016), and while biophysical models are useful to estimate where larvae (and therefore PALs) could potentially migrate, they do not take recruitment success into account. Further, they do not account for genetic spread and/or fixation within populations and are not able to estimate potentially maladapted immigrants that may not survive to adulthood (Marshall, Monro, Bode, Keough, & Swearer, 2010). To better understand the spatial and temporal extent of selective sweeps, key dispersal parameters (effective migration rates and distances between reefs) were incorporated into a single‐locus, two‐dimensional stepping‐stone wave expansion model (Fisher, 1937; Slatkin, 1976) to examine the rate of PAL fixation. Complete fixation (hard sweeps) is one scenario known to elicit phenotypic shifts (Tigano & Friesen, 2016), but it is currently unclear what genetic architecture or specific allele frequencies are needed to result in increased heat tolerance of local populations. Complete fixation was used here as a conservative starting point to assess the spatial extent and timescales for PAL penetrance under natural levels of gene flow and assisted spread scenarios. Wave expansion theory models also incorporate selection coefficients, effective population sizes, mutation rates, and other wave front parameters to provide a more comprehensive quantification of sweeps compared to dispersal kernels (Ralph & Coop, 2010).

**Figure 2:**
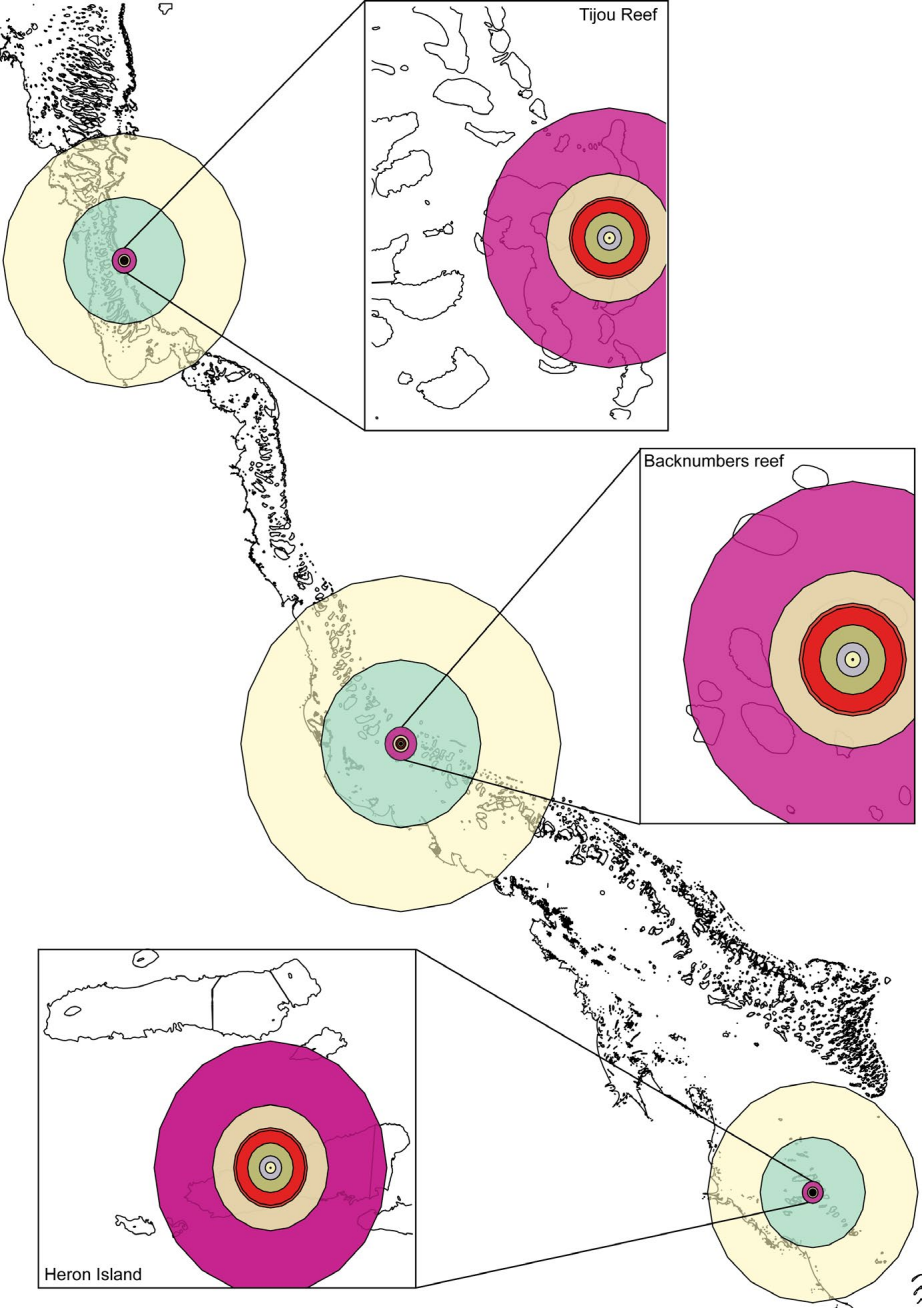
Simulated spread of PAL fixation under a multiple deme stepping‐stone wave expansion model, with the center of variant spread calculated for a northern reef (Tijou reef), central reef (Backnumbers reef) and southern Reef (Heron Island). Colors correspond to different waves of fixation over distinct generation times, from 1 to 1,000 generations of spread. Yellow and teal correspond to 1,000 and 500 generations of variant spread, respectively. Insets for each reef correspond to 1–100 generations of spread (outer most magenta circle: 100 generations, tan circle: 50, dark red: 32, orange red: 30, green: 20‐green, violet: 10, yellow: 5 and pink: 1 generation)

Even using relatively large selection coefficients (0.05 and 0.1), fixation of adaptive variants remained predominantly within the northern reef. Rates derived from Slatkin models demonstrate that with these fixation estimates and a single dispersal origin, the variant wave front would have to travel 7.7 km between reefs in the northern sector, 10.2 km in the central sector, and 13.5 km in the southern sector to reach the next closest reefs for fixation to occur in a single generation, although model outputs estimate fixation after ~50–100 generations (Figure 2a, tan and magenta circles). Fixation with a wave front radius of 67–102.8 km from the three sectors would result in more regional fixation (Figure 2b, teal and yellow circles). Further waves of fixation would radiate outward to other reefs over time, with new wave fronts forming at these reefs as fixation is reached.

The spread of PALs naturally and under assisted scenarios can be described by accelerating, stochastic waves that spread at the leading edge (Slatkin, 1976). The intersection of multiple wave fronts can decelerate sweeps through interference or, alternatively, can accelerate when wave front size is increased over larger areas (i.e., large species ranges; Ralph & Coop, 2010). Hence, theory predicts that sweep velocity can be increased with multiple release sites selected at intervals to avoid the coincidence of wave fronts (Ralph & Coop, 2010). It may therefore be optimal during initial sweep trials to select reefs that are fairly distant to each other in year one and subsequently place variants on reefs that are positioned so their spread will not interfere with wave fronts. Note that these are single variant scenarios, and further complexities arise under multilocus and fixation scenarios and should be incorporated into subsequent models.

### 4.4 How long will natural and assisted fixation of putative adaptive loci take?

Estimating natural rates of PAL fixation will help to determine if active interventions can have a beneficial effect on adaptive rates in local populations. Rapid evolution is possible (<100 years), particularly when species are segregated into metapopulations (Thompson, 1998). Translocation models that simulate genetic rescue demonstrate large improvements in survival probabilities and genetic diversity within 40–50 years of the intervention (Pavlova et al., 2017). For example, rapid improvements in fitness can occur within as little as 10 years, although these estimates are highly dependent on organismal generation times (Pavlova et al., 2017). If fitness benefits are high (i.e., strong selection coefficients exist), then sweeps can occur in as few as 10–100 generations (Coulson et al., 2017; Ferenci, 2008; Hermisson & Pennings, 2017). High selection coefficients can even trump low gene flow and low fitness and lead to fixation, as seen in insecticide resistance development in *Anopheles coluzzii* × *A. gambiae* hybrids (Tigano & Friesen, 2016).

Many corals have comparatively protracted reproductive cycles, with some species taking decades to reach reproductive maturity. However, fast‐growing, branching corals such as the acroporids typically reproduce within two to four years of age (Amar & Rinkevich, 2007; Young, Schopmeyer, & Lirman, 2012). Although they proliferate rapidly, *Acropora* tends to be more thermosensitive compared to slow‐growing massive species (van Woesik, Sakai, Ganase, & Loya, 2011), thereby potentially benefitting most from AGF intervention first. Corals also have overlapping generations, which may initially decrease spread rates through the dilution of the gene pool with non‐PAL carrying individuals, but rates may subsequently increase once multiple generations of PAL containing individuals simultaneously reproduce. Although evolutionary change is considered slower than plastic responses, fast responses may constitute 95% replacement by the advantageous variants after only three generations (Coulson et al., 2017). Encouragingly, beneficial variants need not reach complete fixation to result in an overall positive impact on the receiving populations (Hermisson & Pennings, 2017).

The speed of spread is highly dependent on selection coefficients (*s*), dispersal/migration rates (m) and how population genetic (i.e., deme) structure is modeled (e.g., single continuous, stepping‐stone, or torus; Fisher, 1937; Hartfield, 2012; Morjan & Rieseberg, 2004; Slatkin, 1976). Fisher's wave theory predicts that much of the time needed for the spread of PALs occurs during local fixation in comparison to the time needed to travel between populations (Ralph & Coop, 2010). Furthermore, selection coefficients have a greater effect on the rate of spread (i.e., number of generations necessary for an advantageous mutation to spread across subdivided populations unidirectionally in a stepping‐stone pattern) than organismal migration rates (Morjan & Rieseberg, 2004; Slatkin, 1976). The incorporation of multiple population demes (from 2 to 100) expands the above Fisher wave model structure and results in models that simulate advantageous variants moving through demes in a steplike manner with multiple points of simultaneous expansion (Hartfield, 2012; Slatkin, 1976). Modeling the trajectory of variants and continuous migration over two‐dimensional stepwise space (2D torus; Slatkin, 1976) compared to wave fronts (Fisher, 1937) increases the time to fixation by 14‐fold (Hartfield, 2012). The incorporation of various coefficients is therefore key to understanding the speed of PAL spread.

Using the selection and migration coefficients outlined above, the speed of spread of advantageous variants was estimated using a two or multiple‐dimensional stepping‐stone wave model (Slatkin, 1976) from a single dispersing reef within the northern, central, and southern GBR regions under multiple selection coefficients (Tijou reef, Backnumbers reef, and Heron Island, Figure 2). Adaptive variants took 44–87 generations until fixation was reached when two migrants were introduced into each reef with an effective population size of 500 (*s* = 0.1 and 0.05, respectively). To estimate the rate of spread from a single reef in different regions, the average distance between reefs was estimated by converting rates derived from the Slatkin models to distances, giving 3.38, 4.47, and 2.9 km in the north, central, and southern regions, respectively (see Figure S1). Given the spatial scales within regions of the GBR, the estimated wave velocity rate of fixation of adaptive variants in the north was 0.038–0.077 km per generation, 0.051–0.102 in the central GBR, and 0.033–0.067 in the south for *s* = 0.1 and 0.05. Wave spread rates were slowest in the southern GBR, potentially due to wave interference caused by the smallest average distances between reefs in this sector (Figure 2). These wave rates improve upon earlier models (Fisher, 1937) which estimated fixation would spread at a wave velocity rate between 0.89 and 1.44 km per generation (*s* = 0.1).

Further parameterization of the Slatkin stepping‐stone models also demonstrates the expected slowing behavior (0.06–0.1 km per generation). It would therefore take up to 30–50 generations before variants would travel and reach fixation outside release reefs (red and tan circle, Figure 2) and up to 100 generations to fix at the next proximal reefs (magenta circles, Figure 2). Taking effective population sizes into account in the Slatkin models (*n* = 500) render similar estimates as those derived from 2‐dimensional wave modeling (46 vs. 43.47 generations for fixation; Hermisson & Pennings, 2017). The inclusion of recent effective population size estimates for acroporids (5,000–25,000; Matz et al., 2018) almost doubled this estimate to 78.48 generations to achieve fixation within a single reef (*s* = 0.1). Given these wave velocity rates, the active release of corals should be realized on multiple reefs to increase the rate of spread of PALs over ecologically meaningful time scales, with these release reefs being positioned such that wavefronts will not overlap. Multiple release reefs can also act as insurance against climate variability by having multiple release sites with different climate regimes (“portfolio” or “buffering” effects; Aitken & Whitlock, 2013).

### 4.5 How many individuals are needed for the assisted fixation of putative adaptive loci?

Given these rates of fixation, how many individuals carrying PALs need to be deployed to induce an effective selective sweep (larvae, juveniles or adult colonies) and how many pulses of migrants are needed to sustain PALs in the population? The initial density of variants influences sweep spread rates and some tend to not increase when rare and only when a threshold level is reached or decrease as genetic drift reduces heterozygosity (Pialek & Barton, 1997). Therefore, if too few migrants are used, sweep rates may decrease due to drift. The number of generations needed until fixation decreases rapidly as the number of migrants increases (Morjan & Rieseberg, 2004).

**Figure 3:**
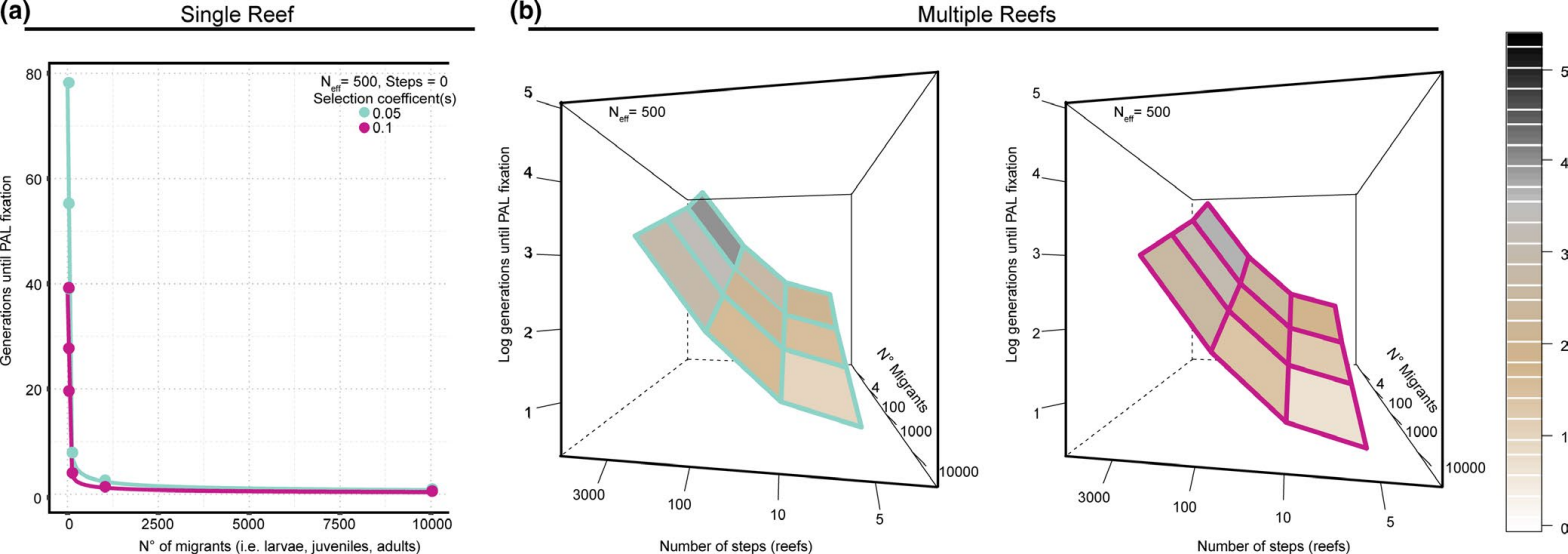
The number of migrants needed to induce fixation on a single reef (a), or multiple reefs (b), under two different selection coefficients (panel 1: *s* = 0.05, panel 2:0.1; teal and pink respectively)

Estimates suggest that going from 1 to 100 migrants would decrease the number of generations until fixation on a single reef from 78 to ~8 (*s* = 0.05) or 39 to 4 (*s* = 0.1), with 1,000 migrants resulting in fixation in about ~1–2.4 generations depending on the selection coefficient (Figure 3a). Increasing the number of reefs to receive migrants with PALs will also increase time to fixation. With a moderate selection coefficient at 0.05, it may take hundreds of generations to reach fixation on multiple reefs if PALs are only released from one reef even if they are released in large numbers (Figure 3b). At *s* = 0.1, 100 reefs could reach fixation within 38 generations if at least 10,000 migrants are used (Figure 3b). Rates of adaptive changes will further vary depending on population‐level genetic diversity, migration rates, phenotypic variance, and selection coefficients (Rehfeldt, Leites, Joyce, & Weiskittel, 2017).

## 5 DISCUSSION

### 5.1 Is AGF feasible given natural recruitment rates?

The addition of hundreds of thousands to millions of corals to the reef is feasible with existing breeding capacity, but high early life mortality rates need to be factored into deployment calculations. Based on previous GBR wide recruitment estimates derived from Hughes et al. (2000), fixation within ~1–2.4 generations may be possible with the previously proposed 1,000 migrants given average estimated recruitment per reef in the north (2,510 ± 774) and central (3,400 ± 1,000) GBR, but may take slightly longer in the south where recruitment appears to be less per reef (774 ± 547; Figure S2a). Given these per reef values, the estimated 10,000 migrants needed to induce fixation through reef restoration and adaptation interventions should be feasible across at least 100 reefs.

This number of migrants is also well within the limits of regional recruitment averages, suggesting ample potential for natural recovery given the estimated spread rates only if the number of migrants is high (Figure S2b). However, the maintenance of this natural recovery potential through changes in allele frequencies may only be possible if adult populations are kept at levels similar to when these censuses were taken (1995–1997; Hughes et al., 2000). Given that mass scale mortality has severely decreased the number of adults in these regions (Hughes et al., 2017), it is likely that natural recovery potential has been severely compromised.

Regardless of the life history stage used, the relatively slow fixation rates, which more generally occur over longer evolutionary timescales, strongly suggest that interventions aimed at increasing the spread of thermal tolerance PALs can have a positive effect on fixation rates for corals of the GBR. Moreover, the biophysical models of larval dispersal using multiple release sites show that some larvae have some capacity to reach further south. If given enough time (hundreds of generations), our stepping‐stone wave models suggest that only four migrants would be needed for fixation to occur if selection is strong, suggesting that even limited larval dispersal may be enough to elicit shifts in allele frequencies, consistent with genetic theory and current modeling (Matz et al., 2018). Furthermore, in order to potentially meet conditions for Hardy–Weinberg equilibrium, stepping‐stone wave models assume non‐overlapping generation times in which each individual is assumed to have only a single generation time. Given that many corals likely have overlapping generations, our values may underestimate fixation rates given non‐overlapping generations may lead to slower fixation as beneficial genes in the incoming population would more quickly be diluted under this assumption. Alternatively, “priority effects” that manifest in species with overlapping generations may slow fixation (Atkins & Travis, 2010; Gilbert et al., 2017). Additional modeling and empirical measurements are required to assess the impact of this assumption as well as determine what allele frequencies (i.e., soft or hard sweeps) are required to illicit increased population survival under thermal stress.

### 5.2 Risks and trade‐offs associated with assisted gene flow

Relatively little is known about the ecological risks associated with AGF and release of ex situ selective bred corals. On a spectrum of translocation actions, AGF within species ranges has been classified as a medium level of intervention action based on the species historical distributions (Seddon, 2010). The formation of outbreeding depression, the breakdown of local adaptation of source populations, and lineage swamping (outcompeting/loss of local sink populations) are the three main risks associated with AGF (reviewed in Aitken & Whitlock, 2013). Other risks may include the depletion of local populations on source reefs or unintentional transfer of pathogens, parasites or members of the microbiome to central or southern reefs. The use of ex situ bred offspring may help partially ameliorate these risks although this approach may introduce other risks associated with the captive propagation approach.

Risks associated with AGF have been quantified as “proportional to the fraction of the population replaced (5%–20%)” and well as the provenance of the coral stock used (Aitken & Whitlock, 2013). Given that little is known about the magnitude of effects in corals, knowledge from other species could be used to predict risk (Kelly & Phillips, 2016). Risk can also be mitigated by limiting the initial numbers of translocated individuals (20% of gene flow in the first pulse, and 2%–4% thereafter; Aitken & Whitlock, 2013). Although estimates of gene flow in corals are limited, the number of migrants per generation may range between 4 and 100 (see above and Matz et al., 2018). Using these estimates, the initial number of translocated individuals needed to mitigate risk associated with assisted PAL spread may equate to roughly 1–20 individuals per reef (20% of 4–100 individuals), which is well below the number needed to achieve fixation within a short time window. Other measures may therefore be needed to help mitigate risk besides limiting the number of individuals used in AGF and deployment of selectively bred corals.

No organism can be perfectly adapted to every environmental pressure, resulting in ecological trade‐offs (Ferenci, 2008; MacArthur & Wilson, 1967). AGF and selective breeding may introduce genotypes that are not adapted to local environmental conditions other than temperature at the receiving location (“local is best”, Rehfeldt et al., 2017) and introduce trade‐offs between different traits. One concern is that the single or repeated pulse of PALs will erode or alter local adaptation among coral populations, as other loci adapted to the source reef conditions will also enter the receiving population (Drury, Manzello, et al., 2017; Drury, Schopmeyer, et al., 2017; Kenkel et al., 2013; Pavlova et al., 2017; Polato et al., 2010; Webber & Scott, 2012). Meta‐analyses across highly divergent taxa including plants, animals, fungi, and protists revealed that local populations gain ~50% fitness advantage in their native environment compared to migrants (Hereford, 2009). Balancing selection may function to decrease this conflict and could be implemented during these intervention practices by selecting populations that are intermediate in their phenotypic ranges (i.e., within ± 1 standard deviation of the phenotypic mean) of those traits of interest (Rehfeldt et al., 2017). The effectiveness of this technique will require the quantification of reaction norms, especially in southern reefs that may be particularly cold‐adapted, likely making the pairing of reefs within ± 1 standard deviation challenging between the most extreme range edges (i.e., northern and southern).

Further considerations involve the ecological outcompeting by source population individuals (genetic swapping/foreign advantage), breakdown of local adaptation, outbreeding depression, and expansion load. When foreigners were placed in different environments compared to their native environment, the presence of foreign advantage was rare among taxa, especially in the circumstances where selection was strong, and where genetic variation was high (Hereford, 2009; Schluter, 2000). Selectively bred organisms (e.g., the offspring of foreign and native individuals) do not always have a competitive advantage over natives (i.e., locals can prevent the establishment of foreigners with higher thermal tolerance; van Oppen et al., 2014; Quigley, Willis, & Bay, 2016); and if present, modeling suggests that this advantage may only be transitory (<2 generations; Aitken & Whitlock, 2013). It has also recently been shown that the addition of foreigners does not lead to as high rates of erosion of local adaptation as originally thought (i.e., decreased rate of sweeps; Tigano & Friesen, 2016). A survey of translocation studies of plants also demonstrate that occurrences of maladaptation caused by outbreeding depression was likely to be low (<3.3%; Leimu & Fischer, 2008). Hence, the risks associated with outbreeding depression and foreign advantage may dissipate rapidly (Aitken & Whitlock, 2013; Harrisson et al., 2016; Ralls et al., 2017; Roitman et al., 2017; Tigano & Friesen, 2016). Finally, expansion load (i.e., the accumulation of deleterious mutations) may slow the spread of genetic variants out of northern populations into the central and southern GBR at the expansion front by removing maladapted populations (Gilbert et al., 2017).

### 5.3 Factors that may increase the efficacy of assisted PAL spread in corals

A number of factors suggest the likelihood of achieving positive fitness benefits for corals through the application of AGF and selective breeding methods is considerable.

1. *Soft sweeps may be sufficient to illicit successful putative adaptive loci penetrance and therefore fitness benefits in corals*. Previous studies have shown that complete fixation may not be necessary to elicit beneficial phenotypic shifts in populations, and allele frequencies as low as 0.2 may be sufficient and a more achievable outcome (Creech et al., 2017; Ferenci, 2008). Therefore, although it is unclear what exact allele frequencies below complete fixation would be sufficient to elicit these shifts, modeling complete fixation provides conservative preliminary estimates. Soft sweeps would also have the secondary benefits of safeguarding against the erosion of genetic diversity and complete elimination of native genetic variants.
2. *Adaptive loci are confirmed in some reefs in the northern Great Barrier Reef and may be present further south*. For AGF or selective breeding to be feasible within the short term, the presence of key standing genetic variation within coral metapopulations is mandatory (pre‐existing PALs). Some adaptive variants involved with thermal tolerance in corals have already been identified in some populations of some coral species and may exist at low frequencies at cooler but warming reefs (Bay & Palumbi, 2014; Dixon et al., 2015; Jin et al., 2016; Louis, Bhagooli, Kenkel, Baker, & Dyall, 2017). Pre‐existing PALs will therefore fast track the spread of adaptive variants throughout populations. Moreover, if PALs already exist, albeit at lower frequencies, in more southern reefs, the combined effects of natural dispersal and with AGF PALs will increase the rate of spread (Hermisson & Pennings, 2017) and potentially decrease the risks of the introduction of maladapted variants. To estimate coefficients of selection, the difficult task of defining “beneficial loci” may be needed (Ferenci, 2008), although it may be initially feasible to use phenotypes to implement AGF and selective breeding. Although substantial progress has been made to characterize the genetic architecture of heat tolerance in corals, it is unclear how many loci are involved, their relative effect sizes, and level of interaction. Furthermore, it is likely that PALs will vary across many reefs in the north which may compete to establish themselves across central and southern reefs and hence slow AGF success.
3. *Naturally high genetic diversity* (Devlin‐Durante & Baums, 2017). High diversity reduces the risk of outbreeding depression, one of the main risks associated with AGF (Aitken & Whitlock, 2013) and leads to faster rates of adaptation (Whiteley, Fitzpatrick, Funk, & Tallmon, 2015). The naturally high genetic diversity in many coral species may thus help to accelerate rates of beneficial fixation. Greater genome diversity (heterozygosity) has also been linked to fitter individuals that exhibit comparatively high resilience (Bay & Palumbi, 2014; Drury, Manzello, et al., 2017; Drury, Schopmeyer, et al., 2017; Ellegren & Galtier, 2016), thereby providing better source material targets for AGF.
4. *Large donor population sizes*. Species with large donor population sizes and wide distributions make good candidates for interventions aimed at increasing PAL spread (Aitken & Whitlock, 2013). Large donor sizes decrease the risk of genetic drift and outbreeding, which can all impede the probability of PALs from reaching high frequencies and can erode adaptive potential (Aitken & Whitlock, 2013; Hereford, 2009). Large donor population sizes also tend to have higher rates of local adaptation (Aitken & Whitlock, 2013; Creech et al., 2017), leading to more efficient selection due to increased genetic diversity (Ellegren & Galtier, 2016; Savolainen, Lascoux, & Merilä, 2013). The potential harnessing of spawning slicks to take advantage of these large population donor sizes may also be feasible (Heyward & Negri, 1999).

## 6 CONCLUSION

This preliminary biophysical modeling suggests that without active intervention, heat tolerance is unlikely to spread beyond the very far northern reefs of the GBR before summer heat waves become annual events. Our biophysical dispersal predictions generally support findings from previous models exploring southern reefs repopulating northern reefs against prevailing currents; however, our model uniquely explores the possibility for potentially bleaching resistant corals to re‐seed central and southern reefs under scenarios of southwards gene flow. Encouragingly, limited connectivity to the central and southern sectors of the GBR is confirmed by our southward biophysical models and by the strong genetic evidence presented in previous studies (Ayre & Hughes, 2004; Lukoschek et al., 2016; van Oppen, Peplow, Peplow, Kininmonth, & Berkelmans, 2011). Our results show that it may take 30+ generations for thermal tolerance PALs to extend beyond single reefs in the far north through natural dispersal, which is too slow to reach central reefs of the GBR in time given IPCC warming estimates. If variants are rare throughout coral populations and only abundant in the far north, 30+ generations to leave one reef may be beyond the scope of time available given that would extend beyond the IPCC 2050 scenario. Modeling at release sites in the central and southern reefs demonstrates that single reef release sites will result in fixation rates that are also too slow. Although rates of adaptive variant spread presented here are based on estimates of selection coefficients and migration alone, our results provide a starting point for further modeling that may include a wider range of parameters, including multilocus, multiallele systems, pleiotropic, and epistatic effects.

Finally, it is currently unclear how variable the genetic architecture of thermal tolerance in corals is. Thus, this single‐locus model provides a reasonable start at modeling changes in allele frequency and fixation of “adaptive” alleles in corals, but additional work is needed to more include multiallele interactions. AGF and selective breeding also involve risk, but they can be managed through the considered selection of source individuals and populations. For example, PAL spread could be limited if a single release site is used or if the site is suboptimal in terms of its physical characteristics that determine larval dispersal away from the reef. Given that the translocation of tens of thousands of individuals may be needed to reach fixation across hundreds to thousands of reefs within ~30 to over 1,000 generations, the translocation of adult colonies without further propagation is not feasible. Instead, ex situ selective breeding of corals for larval or juvenile deployment onto receiving reefs is more practical and achievable approaches to prepare central and southern reefs on the GBR for continued warming.

## CONFLICT OF INTEREST

The authors declare no conflict of interest.

## AUTHOR CONTRIBUTIONS

KMQ, LKB, and MJHO conceived the manuscript. KMQ analyzed the data and wrote the first draft. All authors edited and reviewed the manuscript.

## Data Availability

ConnieII data will be made available from Github (https://github.com/LaserKate).
