# Supplementary material for: The active spread of adaptive variation for reef resilience
Source: Ecol Evol. 2019 Sep 2;9(19):11122–35. doi: 10.1002/ece3.5616 (PMC6802068; doi:10.1002/ece3.5616)
Supplement: Supplementary file 1 [file ECE3-9-11122-s001.docx]

**Supplementary Material**

**Quigley et al.**

**The active spread of adaptive variation for reef resilience**

**Table S1**. Maximum survival in days across 8 *Acropora* species. Full table and references

therein from (Graham et al., 2008).

| Species | Longevity (days) |
| --- | --- |
| *Acropora latistella* | 209 |
| *Acropora millepora* | 110 |
| *Acropora hyacinthus* | 91 |
| *Acropora gemmifera* | 90 |
| *Acropora tenuis* | 69+ |
| *Acropora valida* | 130 |
| *Acropora digitifera* | 45+ |
| *Acropora formosa* | 23 |
| Average | 96 |


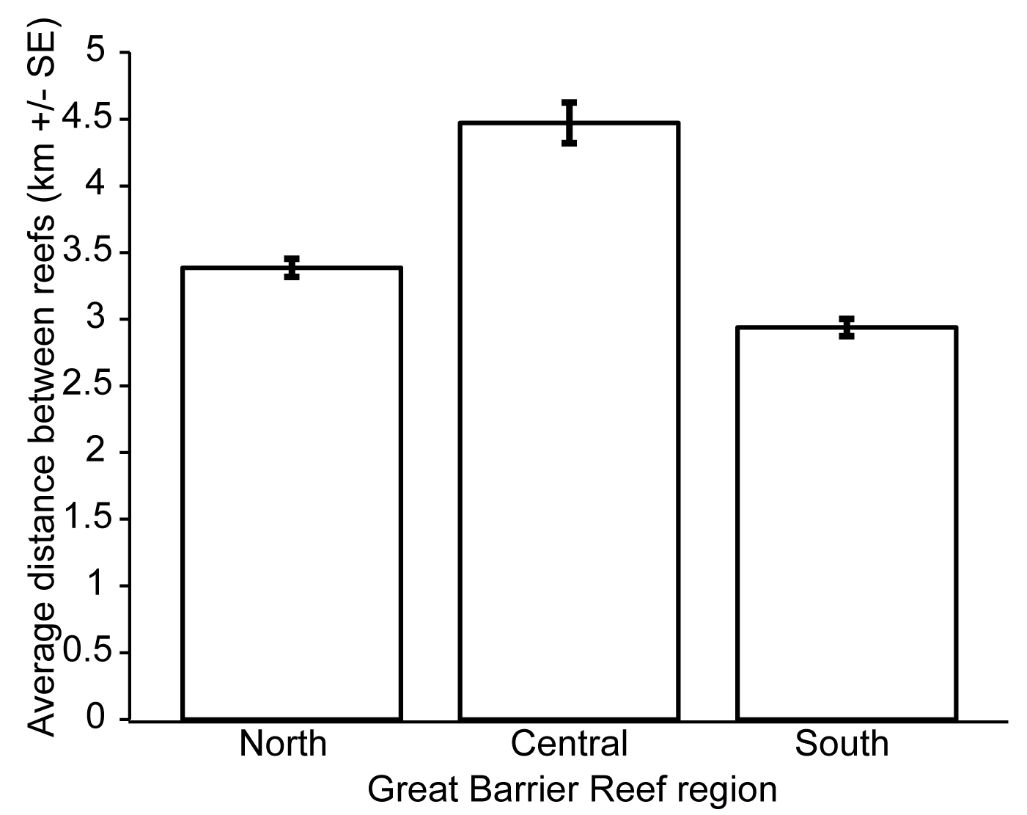


**Figure S1.** Average kilometre distances between reefs per region on the Great Barrier Reef.


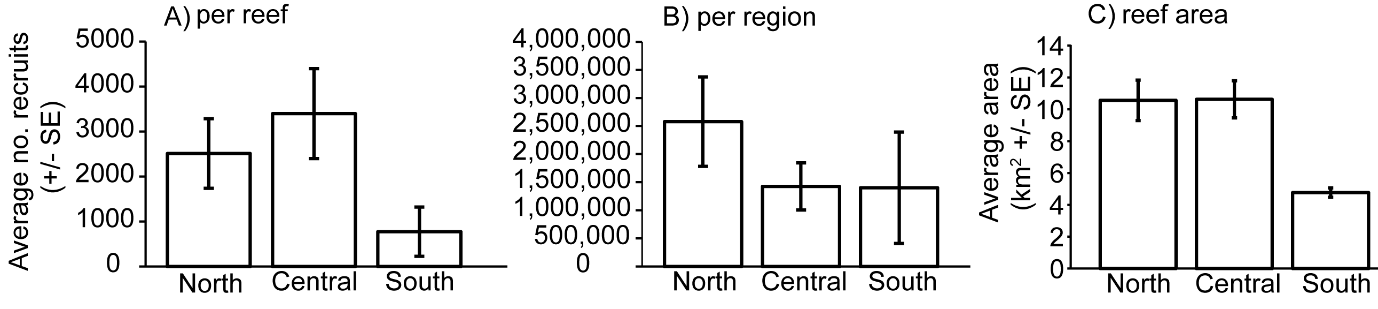


**Figure S2.** Average number of juvenile recruits per reef (A), and per region (B), compared to the average area of reefs per sector (C) from (Hughes et al., 2000; Hughes et al., 2019).

**References**

Graham, E. M., Baird, A. H., & Connolly, S. R. (2008). Survival dynamics of scleractinian coral larvae and implications for dispersal. *Coral Reefs*, *27*(3), 529–539. doi:10.1007/s00338-008-0361-z

Hughes, T. P., Baird, A. H., Dinsdale, E. A., Moltschaniwskyj, N. A., Pratchett, M. S., Tanner, J. E., & Willis, B. L. (2000). Supply-side ecology works both ways: the link between benthic adults, fecundity, and larval recruits. *Ecology*, *81*(8), 2241–2249.

Hughes, T. P., Kerry, J. T., Baird, A. H., Connolly, S. R., Chase, T. J., Dietzel, A., … Woods, R. M. (2019). Global warming impairs stock–recruitment dynamics of corals. *Nature*. doi:10.1038/s41586-019-1081-y
